# Optimal Design of CNT-Nanocomposite Nonlinear Shells

**DOI:** 10.3390/nano10122484

**Published:** 2020-12-10

**Authors:** Leonardo Leonetti, Giovanni Garcea, Domenico Magisano, Francesco Liguori, Giovanni Formica, Walter Lacarbonara

**Affiliations:** 1 CIRTech Institute, Ho Chi Minh City University of Technology (HUTECH), Ho Chi Minh City 725600, Vietnam; 2 Dipartimento di Ingegneria Informatica, Modellistica, Elettronica e Sistemistica, University of Calabria, 8036 Rende, Italy; giovanni.garcea@unical.it (G.G.); domenico.magisano@unical.it (D.M.); francesco.liguori@unical.it (F.L.); 3 Dipartimento di Architettura, University of Roma Tre, 00153 Rome, Italy; giovanni.formica@uniroma3.it; 4 Department of Structural and Geotechnical Engineering, Sapienza University of Rome, 00184 Rome, Italy; walter.lacarbonara@uniroma1.it

**Keywords:** CNT nanocomposite shells, post-buckling optimization, composite optimal design, Koiter method, isogeometry, NURBS interpolation

## Abstract

Carbon nanotube/polymer nanocomposite plate- and shell-like structures will be the next generation lightweight structures in advanced applications due to the superior multifunctional properties combined with lightness. Here material optimization of carbon nanotube/polymer nanocomposite beams and shells is tackled via ad hoc nonlinear finite element schemes so as to control the loss of stability and overall nonlinear response. Three types of optimizations are considered: variable through-the-thickness volume fraction of random carbon nanotubes (CNTs) distributions, variable volume fraction of randomly oriented CNTs within the mid-surface, aligned CNTs with variable orientation with respect to the mid-surface. The collapse load, which includes both limit points and deformation thresholds, is chosen as the objective/cost function. An efficient computation of the cost function is carried out using the Koiter reduced order model obtained starting from an isogeometric solid-shell model to accurately describe the point-wise material distribution. The sensitivity to geometrical imperfections is also investigated. The optimization is carried out making use of the Global Convergent Method of Moving Asymptotes. The extensive numerical analyses show that varying the volume fraction distribution as well as the CNTs orientation can lead to significantly enhanced performances towards the loss of elastic stability making these lightweight structures more stable. The most striking result is that for curved shells, the unstable postbuckling response of the baseline material can be turned into a globally stable response maintaining the same amount of nanostructural reinforcement but simply tailoring strategically its distribution.

## 1. Introduction

Thin-walled, lightweight composite structures are commonly used in a wide range of engineering applications, particularly in aerospace engineering, where they are often employed as primary structural components. Due to the high strength-to-weight ratio, the mechanical response is dominated by buckling and turns out to be mainly influenced by two factors: the geometry and the elastic properties. While the former is often imposed by the structural functionality and only little variations are possible, the spatial distribution of the material properties (e.g., fiber orientations in the layups) can be easily tailored in composite shells. Consequently, an efficient optimization process of the material distribution is required to obtain the desired structural response, usually defined in terms of deflections and load-carrying capacity. Many manufacturing options are also available to fine-tune the stiffness and the onset of buckling: grid stiffeners [1], multi-layered and variable thickness composites [2], variable angle tows (VATs) [3].

A promising direction in the context of material optimization is that offered by nanostructured materials which can exhibit multifunctional properties and are thus prone to more advanced multi-objective optimizations. In this field, nanocomposite materials made of thermosetting or thermoplastic polymers integrated with carbon nanotubes (CNTs) are currently subject to intense developments due to their superior mechanical/electrical/thermal performance, electromagnetic shielding or energy storage capacity [4,5,6,7]. Among other attractive properties exhibited by nanocomposites, a relatively high strength-to-weight ratio, unique damping capability [8,9] and high fatigue tolerance make them ideal candidates for whole new classes of multifunctional composite structures (e.g., high-performance vehicles, aerostructures and devices). Previous works addressed the optimal design of multilayer nanocomposites by fine-tuning the nonlinear interfacial properties regulating the CNT/polymer stick-slip in each of the dedicated layers to achieve maximum storage and damping capabilities [10,11,12]. The optimization problem was restricted to two- and three-layer nanocomposites embedding in selected polymers, CNTs with tunable properties such as the interfacial shear strength and the CNT volume fraction. A robust gradient-free optimization algorithm was developed employing the family of differential evolution optimizers. The objective function was chosen to be the product of the average damping ratio and stored energy of the multilayer nanocomposite within a predefined range of admissible strains.

Moreover, as known, optimal properties in composites are usually sought also by controlling the orientation of the fibers in each layer, since the fiber orientation significantly affects the stiffness distribution, hence, the load-carrying capability or the elastic limits states (see, for instance, [13,14]). Along the same lines, optimization of the CNT distribution within a composite can be achieved to maximize the buckling loads or to change the global stability of nanocomposite shells. Many optimization strategies proposed in the literature use the linearized buckling load as the objective function of the design. However, in this case, structures may suffer another elastic limit state (i.e., static bifurcation) known as buckling mode interaction, which leads to an unstable post-critical behavior [15] and a high sensitivity to imperfections, resulting in a deterioration of their load-bearing capacity due to geometrical, load and material deviations. For this reason, a more reliable design, which takes into account the geometrically nonlinear behavior, has also been investigated in previous works. In this framework, the collapse load of the structure can be defined as the first limit load, for the unstable cases, or as the load magnitudes giving rise to deformations which compromise the usability, taking into account the stiffness reduction that typically characterizes the post-buckling regime. Optimizing the post-buckling behavior in terms of collapse load is, however, a challenging task. In fact, a suitable mechanical model and its discrete approximation are required to describe with acceptable accuracy the geometry, the boundary conditions and the mechanical behavior. This means that the structural response is generally described by a high number of discrete nonlinear equations, whose solution describes the equilibrium path.

The Riks arc-length strategy [16,17] is a standard tool for path following the solutions of a set of nonlinear equations. Although this approach is effective for assigned data, it is not suitable for an optimization process, which requires the evaluation of the equilibrium path for any change in the design variables, and for an imperfection sensitivity analysis, because a single run is too time-consuming with current CPUs. Promising generalizations of the path following strategy have been presented [18,19,20] with the aim of performing parametric studies in a more efficient way.

In the optimization strategy presented in [21], the collapse load is evaluated by a nonlinear finite element (FE) buckling problem. The algorithm is extended in [22,23] in order to take into account the worst geometrical imperfection case. An interesting way of analyzing slender structures is offered by strategies based on Koiter’s theory of elastic stability [24]. They make use of an asymptotic expansion of the equilibrium equations which allows the description of the initial post-critical behavior in terms of some variables related to the slope and curvature of the bifurcated branches [25].

More recently, a solution algorithm based on Koiter’s theory implemented within a Finite Element environment was proposed in [26,27]. It allows structures to be optimized with general geometries, loading and boundary conditions. Moreover, the strategy exhibits good levels of accuracy in predicting the initial postbuckling response of several structures due to a multi-modal asymptotic expansion which accounts also for nonlinear buckling modal interactions [28]. The strategy is also capable of efficiently providing the worst equilibrium path through a statistical estimation of the worst-case imperfection, which is assumed to belong to the space of the buckling modes of the structure under consideration. A hybrid solution strategy, referred to as the Koiter-Newton approach, was further investigated in [29,30].

Despite the difficulties associated with the prediction of the nonlinear behavior, another challenge is the solution to the optimization problem. This is always expressed as a nonlinear, nonconvex mathematical programming problem whose solution is generally computationally expensive and extremely difficult because of the likely presence of multiple local minima. This is indeed the most penalizing aspect of the analysis. Among others, frequently employed solution strategies are the random search methods [31], genetic algorithms [32] and gradient-based techniques such as the method of moving asymptotes [33] or sequential linear programming [34].

This work addresses the optimization problem of nanocomposite shell-like structures with variable CNTs distributions. Three types of optimizations are tackled: (a) through-the-thickness CNT distribution in terms of volume fractions, (b) randomly oriented CNT distributions across the mid-surface in terms of volume fraction, (c) distribution of aligned CNTs across the mid-surface in terms of orientation. The collapse load, including limit points and deformation limits, is taken as the objective function. Its efficient estimate is carried out using a reduced order model (Koiter’s method) obtained starting from an isogeometric solid-shell model capable of accurately modeling the variable material distribution. The sensitivity to geometrical imperfections is also addressed as in [26]. The optimization is performed using the Global Convergent Method of Moving Asymptotes (GCMMA). A numerical investigation is carried out to assess how varying the volume fraction distribution, as well as the CNTs orientation, affects the stability of nanocomposite shells. It is shown that an unprecedented tuning of the shells stability can be achieved in different ways, either affecting the local bifurcation behavior (e.g., shifting the buckling loads to higher values) or by affecting the global behavior (e.g., suppressing the snap-through instability).

The paper is organized as follows: Section 2 describes the solid-shell model for elastic shell structures; Section 3 introduces the isogeometric NURBS-based discretization technique; the constitutive equations for a polymeric matrix reinforced with CNTs are reported in Section 4; Section 5 formulates the nonlinear optimization problem for variable distributions of CNTs in the shell domain; the numerical optimization strategy based on Koiter method and GCMMA is presented in Section 6; extensive numerical analyses are carried out in Section 7; conclusions are drawn in Section 8.

## 2. Solid-Shell Model

This section describes the main equations of the solid-shell FE formulation (see [35]) used to construct the discrete model. The outset is the 3D Cauchy continuum whose geometry of deformation is described by the Green–Lagrange strains. By employing a solid-shell concept, a linear through-the-thickness interpolation is assumed for the kinematic unknowns. Euclidean vectors will be denoted by boldface, italic, lower case letters, algebraic vectors in $R^{3}$ will be denoted by boldface, non-italic letters; tensors by boldface, italic, upper case letters, matrices by boldface, non-italic, upper case letters.

### Stored and Complementary Energy

Convective curvilinear shell coordinates $\zeta =(\zeta_{1},\zeta_{2},\zeta_{3})$ are employed, with $\left(\zeta_{1},\;\zeta_{2}\right)$ representing mid-surface coordinates and $\zeta_{3}\in \left[-\frac{h}{2},\frac{h}{2}\right]$ being the thickness coordinate with *h* the shell thickness. The position vector p(ζ) of material points in the current configuration is given in terms of their position vector x(ζ) in the reference configuration and the displacement u(ζ),

(1) p(ζ)=x(ζ)+u(ζ).

Note that while (x,p,u) are treated as Euclidean vectors, ζ is a vector of $R^{3}.$ The covariant basis vectors in the undeformed configuration are obtained from the corresponding partial derivatives $G_{i}=x{,}_{i}$ of the position vectors x, where $\left(\right){,}_{i}$ indicates partial differentiation with respect to the *i*-th component of ζ. By letting $G^{i}$ denote the contravariant basis so that $G_{i}\cdot G_{j}=\delta_{i}^{j}$ with $\delta_{i}^{j}$ the Kronecker delta and (·) the dot product, the Green–Lagrange strain tensor can be expressed as

(2) $$E={\bar{E}}_{ij}\;G^{i}⊗G^{j},\;{\bar{E}}_{ij}=\frac{1}{2}\left(x{,}_{i}\cdot u{,}_{j}+u{,}_{i}\cdot x{,}_{j}+u{,}_{i}\cdot u{,}_{j}\right),$$

where (⊗) indicates the tensor product.

Assuming a linear through-the-thickness interpolation, the position vector is expressed as

(3) $$x\left(\zeta \right)=x_{0}\left(\zeta_{1},\zeta_{2}\right)+\frac{2\zeta_{3}}{h}x_{n}\left(\zeta_{1},\zeta_{2}\right)$$

where $x_{0}:=\frac{1}{2}\left(x\left(\zeta_{+}\right)+x\left(\zeta_{-}\right)\right)$ and $x_{n}:=\frac{1}{2}\left(x\left(\zeta_{+}\right)-x\left(\zeta_{-}\right)\right)$, with $\zeta_{+}=\left(\zeta_{1},\zeta_{2},\frac{h}{2}\right)$ and $\zeta_{-}=\left(\zeta_{1},\zeta_{2},-\frac{h}{2}\right)$. Similarly, the displacement field is described as

(4) $$u=u_{0}\left(\zeta_{1},\zeta_{2}\right)+\frac{2\zeta_{3}}{h}u_{n}\left(\zeta_{1},\zeta_{2}\right)$$

with $u_{0}:=\frac{1}{2}\left(u\left(\zeta_{+}\right)+u\left(\zeta_{-}\right)\right)$ and $u_{n}:=\frac{1}{2}\left(u\left(\zeta_{+}\right)-u\left(\zeta_{-}\right)\right)$ being the coordinates of the upper and lower surfaces of the shell. The independent strain components in Equation (2) are collected in the six-dimensional strain vector $\epsilon ={\left[E_{11},E_{22},2E_{12},E_{33},2E_{23},2E_{13}\right]}^{T}$ and linearized with respect to $\zeta_{3}$ as

(5) $$\epsilon \approx \left[\begin{array}{c} e\left(\zeta_{1},\zeta_{2}\right)+\zeta_{3}\;\chi \left(\zeta_{1},\zeta_{2}\right)\\ E_{33}\left(\zeta_{0}\right)\\ \gamma (\zeta_{1},\zeta_{2}) \end{array}\right]$$

with $\zeta_{0}=\left(\zeta_{1},\zeta_{2},0\right)$ and the membrane strain vector e, the curvature vector χ, and the transverse shear strains vector γ given by

$$e\left(\zeta_{1},\zeta_{2}\right)=\left[\begin{array}{c} E_{11}\left(\zeta_{0}\right)\\ E_{22}\left(\zeta_{0}\right)\\ 2E_{12}\left(\zeta_{0}\right) \end{array}\right],\;\chi \left(\zeta_{1},\zeta_{2}\right)=\left[\begin{array}{c} E_{11}{,}_{3}\left(\zeta_{0}\right)\\ E_{22}{,}_{3}\left(\zeta_{0}\right)\\ 2E_{12}{,}_{3}\left(\zeta_{0}\right) \end{array}\right],\;\gamma \left(\zeta_{1},\zeta_{2}\right)=\left[\begin{array}{c} 2E_{23}\left(\zeta_{0}\right)\\ 2E_{13}\left(\zeta_{0}\right) \end{array}\right].$$

The constitutive equations, in Voigt notation, of the 3D continuum are assumed to be those of a transversally isotropic material with the axis of transverse isotropy collinear with the axis of the CNTs. Hence, they can be rewritten as the following block matrix:

$$L=\left[\begin{array}{ccc} L_{m} & 0 & 0\\ 0 & L_{33} & 0\\ 0 & 0 & L_{s} \end{array}\right],$$

by neglecting the coupling between the membrane strains e and the thickness strain $E_{33}$ and blocks defined by Equation (5). The block matrix $L_{s}$ describes the shear elastic constants. The membrane-related block matrix is evaluated assuming plane stress conditions in order to avoid thickness locking, while the transverse elastic constant $L_{33}$ is maintained to avoid zero-energy modes (thickness stretch).

By denoting with *V* the region occupied by the shell in the reference configuration and Ω the area of its mid-plane and performing the integration over the thickness, we obtain

(6) $$\int_{V}\epsilon^{⊤}L\epsilon dV=\int_{\Omega }\int_{-h/2}^{h/2}\epsilon^{⊤}L\epsilon d\zeta_{3}d\Omega =\int_{\Omega }\varepsilon^{⊤}C\varepsilon d\Omega ,$$

where vector $\varepsilon \left(\zeta_{1},\zeta_{2}\right):={\left[e^{⊤},E_{33},\chi^{⊤},\gamma^{⊤}\right]}^{⊤}$ collects the generalized strains and C is the generalized constitutive matrix expressed as

(7) $$C=\left[\begin{array}{cccc} C_{ee} & 0 & C_{e\chi } & 0\\ 0 & C_{33} & 0 & 0\\ C_{e\chi }^{T} & 0 & C_{\chi \chi } & 0\\ 0 & 0 & 0 & C_{\gamma \gamma } \end{array}\right]$$

with

$$C_{ee}=\int_{-\frac{h}{2}}^{\frac{h}{2}}L_{m}d\zeta_{3},\;C_{e\chi }=\int_{-\frac{h}{2}}^{\frac{h}{2}}\zeta_{3}L_{m}d\zeta_{3},\;C_{\chi \chi }=\int_{-\frac{h}{2}}^{\frac{h}{2}}\zeta_{3}^{2}L_{m}d\zeta_{3},$$

$$C_{33}=\int_{-\frac{h}{2}}^{\frac{h}{2}}L_{33}d\zeta_{3},\;C_{\gamma \gamma }=\int_{-\frac{h}{2}}^{\frac{h}{2}}L_{s}d\zeta_{3}.$$

## 3. Isogeometric Solid-Shell Model

The continuum solid-shell model is discretized by using NURBS functions. In particular, according to IGA, the same interpolation is used for the geometry and displacements [35,36].

**NURBS basics**. A B-Spline curve is represented as

(8) $$g\left(\zeta \right)=\sum_{i=1}^{n}N_{i}^{p}\left(\zeta \right)y_{i}=N\left(\zeta \right)\cdot y,$$

where $y_{i}$ (i=1⋯n) are control points and $N_{i}^{p}\left(\zeta \right)$ are the set of B-Spline basis functions taken as piecewise polynomial functions of order *p*. The latter are defined by a set of nondecreasing real numbers $\Xi =[\zeta^{1},\zeta^{2},\;\cdots ,\;\zeta^{n+p+1}]$ known as knot vectors. B-Spline basis functions are calculated recursively by using

(9) $$N_{i}^{p}\left(\zeta \right)=\frac{\zeta -\zeta^{i}}{\zeta^{i+p}-\zeta^{i}}N_{i}^{p-1}\left(\zeta \right)+\frac{\zeta^{i+p+1}-\zeta }{\zeta^{i+p+1}-\zeta^{i+1}}N_{i+1}^{p-1}\left(\zeta \right),$$

for p≥1 and starting from piecewise constant functions (p=0) defined as

$$N_{i}^{0}\left(\zeta \right)=\left\{\begin{array}{cc} 1 & ,\;\;\;\;\mathrm{if}\;\;\;\zeta^{i}\leq \zeta \leq \zeta^{i+1}\\ 0 & ,\;\;\;\;\mathrm{otherwise}. \end{array}\right.$$

The regularity of B-Spline basis functions is given by r=p−s, where *p* and *s* are the order used for the basis functions and the multiplicity of the knot $\zeta_{i}$, respectively. NURBS functions are obtained by a projective transformation of the B-splines by extending Equation (8) with the following shape functions:

(10) $$R_{i}^{p}\left(\zeta \right)=\frac{N_{i}^{p}\left(\zeta \right)w_{i}}{\sum_{i}^{n}N_{i}^{p}\left(\zeta \right)w_{i}}.$$

It is worth noting that all properties of B-Splines are retained and, in particular, a B-Spline is retrieved when all weights are equal.

By applying the tensor product, the NURBS surface is constructed in a way similar to Equation (8) as

(11) $$q\left(\zeta_{1},\zeta_{2}\right)=\sum_{i=1}^{n}\sum_{j=1}^{m}R_{i}^{p}\left(\zeta_{1}\right)M_{j}^{q}\left(\zeta_{2}\right)Y_{ij}=N\left(\zeta_{1},\zeta_{2}\right)Y,$$

where $R=[\zeta_{1}^{1},\zeta_{1}^{2}\cdots \zeta_{1}^{n+p+1}]$ and $M=[\zeta_{2}^{1},\zeta_{2}^{2}\cdots \zeta_{2}^{m+q+1}]$ are two knot vectors, $R_{i}^{p}$ and $M_{j}^{q}$ are the one-dimensional basis functions over them and $Y_{ij}$ defines a set of n×m control points. The tensor product between the knot vectors R and M defines a mesh of quadrilateral *isogeometric elements*.

Weights, as well as control points of the initial geometry, are provided by the CAD model while suitable algorithms exist for the refinement required to approximate the unknown solution [37]. The geometry is represented exactly regardless of the adopted mesh.

**Isogeometric interpolation**. In this subsection, the discrete isogeometric model used within the optimization strategy is summarized. The geometry is described by NURBS interpolation functions as

(12) $$x\left(\zeta \right)=N_{u}\left(\zeta \right)x_{e}$$

where $x_{e}=\left[x_{0e},x_{ne}\right]$ collects the control points of the geometry corresponding to $x_{0}$ and $x_{n}$, respectively. The matrix $N_{u}\left(\zeta \right)$ collects the interpolation functions

(13) $$N_{u}\left(\zeta \right):=\left[\begin{array}{c} N\left(\zeta_{1},\zeta_{2}\right),\frac{2\zeta_{3}}{h}N\left(\zeta_{1},\zeta_{2}\right) \end{array}\right]$$

where $N(\zeta_{1},\zeta_{2})$ are bi-dimensional NURBS (11) functions of the mid-surface coordinates only.

By following the isogeometric concept, the displacement field is interpolated using the same shape functions of the geometry

(14) $$u\left(\zeta \right)=N_{u}\left(\zeta \right)u_{e}\;$$

where $u_{e}=\left[u_{0e},u_{ne}\right]$ collects the control points for the displacement fields $u_{0}$ and $u_{n}$.

The Green–Lagrange strains in Equation (5), upon considering Equations (12) and (14), become

(15) $$\varepsilon \left(\zeta_{1},\zeta_{2},u_{e}\right)=\left(L\left(\zeta_{1},\zeta_{2}\right)+\frac{1}{2}Q\left(\zeta_{1},\zeta_{2},u_{e}\right)\right)u_{e},$$

where $L\left(\zeta_{1},\zeta_{2}\right):=Q\left(\zeta_{1},\zeta_{2},x_{e}\right)$ and $Q(\zeta_{1},\zeta_{2},u_{e})$ has a linear dependence from $u_{e}$ and its expression can be found in [35].

**Stored energy and equilibrium path**. The stored energy of the shell can be evaluated using a numerical integration as

(16) $$\Phi =\sum_{e}\Phi_{e},\;\Phi_{e}=\frac{1}{2}\sum_{g}\left(\varepsilon_{g}{\left(u_{e}\right)}^{⊤}C_{g}\varepsilon_{g}\left(u_{e}\right)\right)w_{g},$$

where *e* denotes the FE, *g* indicates the integration point and $w_{g}$ is the corresponding weight. By taking advantage of the high continuity of the NURBS functions, patch-wise integration schemes can be adopted, thereby reducing the number of integration points. Moreover, well-tuned patch-wise reduced scheme can avoid locking (for more details on these aspects, see [35,36]).

The system of discrete equilibrium equations is then obtained through enforcement of the stationarity of the total potential energy according to

(17) $$r\left(\lambda ;u\right)=\frac{\partial \Phi }{\partial u}-\lambda \bar{f}=n\left(u\right)-\lambda \bar{f}=0,$$

where r is the residual vector, n(u) is the vector of generalized stress resultants (i.e., restoring forces), $\bar{f}$ is the load vector per unit multiplier, u collects the discrete displacement variables of the isogeometric model and λ is the load multiplier. The solutions of Equation (17) define the equilibrium paths of the structure in the u−λ space.

## 4. Constitutive Formulation for CNT Nancomposite Shells

**Figure 1:**
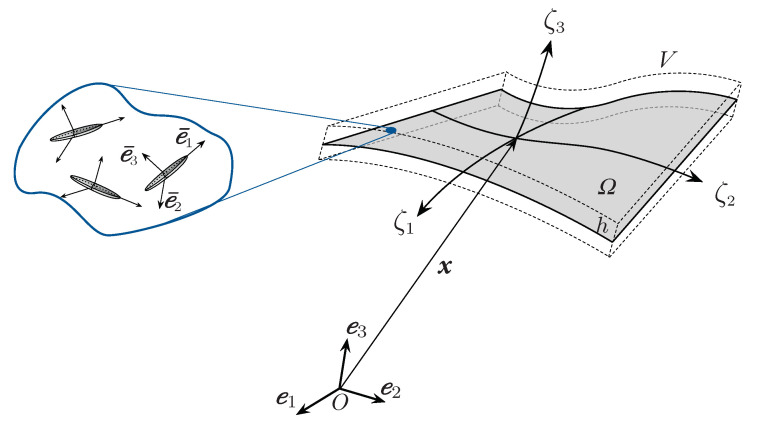
Schematic geometry of the carbon nanotube (CNT) nanocomposite shell.

Our aim is to tailor the nanostructured material distribution within the shell that leads to the optimization of a certain property of the nonlinear equilibrium path obtained (e.g., maximizing the collapse load) by solving the equilibrium problem later expressed by Equation (27). Before delving into further details of the material tailoring problem, we pause to discuss the constitutive model for nanocomposites with randomly oriented or perfectly aligned CNTs (Figure 1).

A compact expression of the effective elastic tensor for the constitutive law of the 3D continuum is given by

(18) $$\bar{L}=L_{M}+\phi_{C}\langle B\rangle {\left[\left(1-\phi_{C}\right)I+\phi_{C}\langle A\rangle \right]}^{-1}$$

with

$$B=〚L〛A,\;〚L〛=L_{C}-L_{M},\;A={\left(I+SL_{M}^{-1}〚L〛\right)}^{-1},$$

where I is the identity tensor, $L_{C}$ and $L_{M}$ are the elastic tensors of the CNT inclusions and the matrix, respectively, 〚L〛 is the elastic mismatch, and S is the Eshelby tensor.

Tensor B must be transformed in order to account for a generic orientation of the material frame $\left\{{\bar{e}}_{1},{\bar{e}}_{2},{\bar{e}}_{3}\right\}$ according to

(19) $${\bar{B}}_{ijkl}=c_{ip}c_{jq}c_{kr}c_{ls}B_{pqrs}$$

where the explicit expression of the transformation coefficients $c_{ip}$ is reported in [13]. Subsequently, the notation 〈·〉 indicates an averaging over the range of the CNTs orientations and their expression is given in [13]. Two cases are considered here:

(i) randomly orientated CNTs
  $$\langle B\rangle =\frac{1}{8\pi^{2}}\int_{0}^{2\pi }\int_{0}^{\pi }\int_{0}^{2\pi }\bar{B}\sin\vartheta d\varphi d\vartheta d\beta$$
(ii) CNTs alignment along ${\bar{e}}_{1}$
  $$\langle B\rangle =\frac{\int_{0}^{2\pi }\int_{0}^{\pi }\int_{0}^{2\pi }\bar{B}f\left(\varphi ,\beta \right)\sin\vartheta d\varphi d\vartheta d\beta }{\int_{0}^{2\pi }\int_{0}^{\pi }\int_{0}^{2\pi }f\left(\varphi ,\beta \right)\sin\vartheta d\varphi d\vartheta d\beta },$$

where (φ,ϑ,β) are the Euler angles providing the CNTs orientation with respect to a fixed frame, f(φ,β) is the orientation distribution function which, for the case of fibers aligned along the ${\bar{E}}_{1}$ axis, becomes f(φ,β)=δ(φ−0)δ(β−0) (see [13,14] for details).

The constitutive relation of the shell is obtained as $L=R\bar{L}R^{⊤}$ where R=R(θ) is the rotation matrix with θ indicating the angle between ${\bar{e}}_{1}$ and the $e_{1}$ axis of the shell, while $e_{3}:={\bar{e}}_{3}$.

It is also possible to enhance the accuracy of the Eshelby–Mori–Tanaka model by taking into account the actual CNTs aspect ratio and the CNTs macrodispersion in the considered nanocomposites [14].

## 5. Postbuckling Optimization of CNT Nanocomposite Shells

Several types of material optimizations will be sought, namely, (1) through-the-thickness optimization of the aligned CNTs volume fraction; (2) optimization of randomly orientated CNTs volume fraction; (3) optimization of the in-plane CNTs orientation. We will discuss each of these problems next.

### 5.1. Through-the-Thickness Optimization of the Aligned CNTs Volume Fraction

In the first case, the optimal distribution of CNTs parameters is obtained by changing the through-the-thickness CNTs distribution across the shell mid-surface.

The volume fraction of CNTs is assumed to be distributed as

$$\phi_{C}\left(\zeta \right)=\left[1+\frac{2}{h}a\left(\zeta_{1},\zeta_{2}\right)\zeta_{3}\right]\phi_{C}^{*},$$

which means that the through-the-thickness average $\phi_{C}^{*}$ of $\phi_{C}\left[\zeta \right]$ is constant over the shell domain, while $a(\zeta_{1},\zeta_{3})$ is a function of the through-the-thickness distribution such that $-1\leq a(\zeta_{1},\zeta_{2})\leq 1$. In this way, we limit the volume fraction variability to lie in the range $0\leq \phi_{C}\leq 2\phi_{C}^{*}$. The constitutive matrix $L=\bar{L}$ is that obtained for the case of uniformly aligned CNTs. The thickness-wise variability function $a(\zeta_{1},\zeta_{2})$ can be described using Bernstein polynomials over the whole shell with discrete parameters a:

(20) $$a\left(\zeta_{1},\zeta_{2}\right)=N_{a}\left(\zeta_{1},\zeta_{2}\right)a.$$

By simply increasing the polynomial order, more complex material distributions can be obtained. Considering a certain feature P[a] of the shell response, its minimization is expressed as

(21) $$\begin{array}{cccc} \; & \underset{a}{\mathrm{minimise}}\; & \; & P[a]\\ \; & \mathrm{subject}\;\mathrm{to}\; & \; & -1\leq N_{a}\left(\zeta_{1g},\zeta_{2g}\right)a\leq 1\;\forall g. \end{array}$$

### 5.2. Optimization of Randomly Orientated CNTs Volume Fraction

The optimal distribution of CNTs in this instance is obtained by changing the volume fraction $\phi_{C}\left(\zeta_{1},\zeta_{2}\right)$ over the mid-surface of the shell, while maintaining it constant through the thickness direction $\zeta_{3}$. For this optimization problem, all parameters describing the domain variation of the CNTs volume fraction $\phi_{C}\left(\zeta_{1},\zeta_{2}\right)$ are collected in vector ϕ and interpolated as

$$\phi_{C}\left(\zeta_{1},\zeta_{2}\right)=N_{\phi }\left(\zeta_{1},\zeta_{2}\right)\phi .$$

As in the previous case, the CNTs volume faction distribution at a point of the domain can be obtained using Bernstein polynomials throughout the whole shell. The constitutive matrix $L=\bar{L}$ is that obtained for the randomly oriented CNTs.

The minimization problem is cast in the form

(22) $$\begin{array}{cccc} \; & \underset{\phi }{\mathrm{minimize}}\; & \; & P(\phi )\\ \; & \mathrm{subject}\;\mathrm{to}\; & \; & \phi_{C}^{min}\leq N_{\phi }\left(\zeta_{1g},\zeta_{2g}\right)\phi \leq \phi_{C}^{max}\;\forall g\\ \; & \; & & V_{C}=h\int_{\Omega }\phi_{C}d\Omega ={\bar{V}}_{C} \end{array}$$

where the point-wise value $\phi_{C}$ is constrained to vary between the upper bound $\phi_{C}^{max}$ and the lower bound $\phi_{C}^{min}$ and the total CNT volume is set to the fixed value ${\bar{V}}_{C}$.

### 5.3. Optimization of the in-Plane CNTs Orientation

The CNTs are assumed to be aligned along a direction parallel to the shell mid-surface at an angle θ with respect to $e_{1}$. The constitutive matrix at each point of the shell is $L=R\bar{L}R^{⊤}$ with the rotation matrix R=R(θ), with θ being constant through the thickness, and the constitutive matrix $\bar{L}$ obtained for the aligned case. The spatial distribution of orientation angles $\theta (\zeta_{1},\zeta_{2})$ is described using Bernstein polynomials over the whole shell. The angle distribution can be expressed as

$$\theta \left(\zeta_{1},\zeta_{2}\right)=N_{\theta }\left(\zeta_{1},\zeta_{2}\right)\vartheta .$$

upon collecting the discrete angle parameters in the vector ϑ.

The optimization problem reads

(23) $$\begin{array}{cccc} \; & \underset{\vartheta }{\mathrm{minimize}}\; & \; & P(\vartheta )\\ \; & \mathrm{subject}\;\mathrm{to}\; & \; & \frac{-\pi }{2}\leq N_{\theta }\left(\zeta_{1g},\zeta_{2g}\right)\vartheta \leq \frac{\pi }{2}\;\forall g \end{array}$$

## 6. Objective Function and Optimization Algorithm

**The objective function**. The optimization process is aimed at maximizing the collapse load of the nanocomposite shells. In buckling problems, the collapse load can be defined as the lower bound between the critical limit load $\lambda_{lim}$ and the load associated with a deformation limit $\lambda_{def}$. We denote with α the vector collecting the generic design optimization parameters. In this work, α coincides with the material variables: either a, ϕ or ϑ introduced in the previous section for the three stated optimization problems, respectively. The objective function can thus be written as

(24) $$P\left(\alpha \right)=-\lambda_{c}=-min\left(\lambda_{lim},\lambda_{def}\right).$$

The evaluation $\lambda_{lim}$ and $\lambda_{def}$, and thus the objective function computation, requires the construction of the equilibrium path of the structure for assigned design variables α. A common approach for path following the equilibrium curve is the Riks arc-length method [16,35]. In this case, the nonlinear equations in the kinematic unknowns are solved step-by-step using the Newton-Raphson method. However, this kind of analysis bears a significant computational cost due to the large size of the matrices associated with a the high number of DOFs. Furthermore, a reliable evaluation of the equilibrium path should take into account the sensitivity of the structure to imperfections, in particular, to geometrical imperfections. In this case, the nonlinear analysis has to be repeated for a large number of imperfection shapes [26,27], in order to detect the worst imperfection scenario [22]. For this reason, in this work we use an alternative approach called Koiter’s method [25]. Hence, a reduced order model based on Koiter’s theory of elastic stability is assembled for the assigned material configuration. Then, the corresponding reduced nonlinear equations, usually in a lower number of unknowns, are solved to obtain a good estimate of the equilibrium path with the benefit of a much lower computational cost. Moreover, the most interesting feature of the method is the possibility of obtaining the equilibrium path of the imperfect structure by including imperfections a-posteriori in the reduced system [28] of the perfect structure, thus enabling inexpensive sensitivity analyses.

**Koiter’s method**. The structure is first discretized using the isogeometric environment described in Section 3. Then, the stored energy of each element is rewritten in a mixed form using the stresses at each integration point $\sigma_{g}$ as independent variables [17]

(25) $$\Phi_{e}=\sum_{g}\left(\sigma_{g}^{T}\varepsilon_{g}\left(u_{e}\right)-\frac{1}{2}\sigma_{g}^{T}C_{g}^{-1}\left(\alpha \right)\sigma_{g}\right)w_{g}$$

As shown in [36], this is necessary to improve Koiter’s method accuracy, because of the direct prediction of the stress, and efficiency, due to the vanishing of the forth order strain energy variations.

By collecting in vector z the global discrete displacements u and stresses $\sigma_{g}$ at each integration point *g*, Koiter’s method is based on the following reduced order model:

(26) $$z\left(\lambda ;\psi_{i}\right)=\lambda \bar{z}+\sum_{i=1}^{m}\psi_{i}{\dot{v}}_{i}+\frac{1}{2}\sum_{i,j=1}^{m}\psi_{i}\psi_{j}w_{ij}+\frac{1}{2}\lambda^{2}\hat{\hat{w}}$$

where $\psi_{i}$ are the scalar values defined as modal amplitudes, $\hat{z}$ is the linear elastic solution (path tangent to the stress-free configuration), ${\dot{v}}_{i}$ denotes the *i*th of the *n* buckling modes, $w_{ij}$ and $\hat{\hat{w}}$ are quadratic corrections. The evaluation of these vectors requires the solution of linear systems for $\hat{z}$, $w_{ij}$ and $\hat{\hat{w}}$ and a linearized buckling analysis for ${\dot{v}}_{i}$. Details can be found in [25,36].

According to this choice and after a few asymptotic expansions, it is possible to obtain the following nonlinear algebraic reduced system of equilibrium equations where the unknowns are $\psi_{i}$ and λ

(27) $$\begin{array}{cc} r_{k}\left[\lambda ,\psi_{1},\cdots ,\psi_{m}\right] & =\mu_{k}\left[\lambda \right]+\left(\lambda_{k}-\lambda \right)\psi_{k}-\frac{1}{2}\lambda^{2}\sum_{i=1}^{m}\psi_{i}C_{ik}+\frac{1}{2}\sum_{i,j=1}^{m}\psi_{i}\psi_{j}A_{ijk}\\ \; & +\frac{1}{6}\sum_{i,j,h=1}^{m}\psi_{i}\psi_{j}\psi_{h}B_{ijhk}=0,\;k=1\cdots m \end{array}$$

The coefficients $A_{ijk}$, $C_{ik}$, $B_{ijhk}$ and $\mu_{k}\left[\lambda \right]$ are scalar quantities evaluated as the sum of elemental contributions of the stored energy variations. Their explicit expressions can be found in [25,36].

A remarkable advantage afforded by Koiter’s method is the possibility of performing computationally efficient and robust imperfection sensitivity analysis of elastic structures. Once Equation (27) has been solved for the perfect structure, the imperfect structure can be studied by only perturbing a posteriori the same system, or rather by adding to it the imperfection term ${\tilde{\mu }}_{k}$. Therefore, the system in Equation (27) becomes

(28) $$r_{k}+{\tilde{\mu }}_{k}=0,\;k=1\cdots m.$$

This means that all coefficients of (28) coincide with those evaluated for the perfect structure and thus the analysis of the effect of a new geometrical imperfection simply requires to update ${\tilde{\mu }}_{k}$ and solve again Equation (28). In this fashion, it is possible to test thousands of imperfections in a few seconds. It is worth noting that, for a given structure, it is impossible to obtain the same amount of results in such a short time if the nonlinear analysis is performed with the full discrete model.

For the evaluation of ${\tilde{\mu }}_{k}$, two strategies were previously proposed [28]. A first solution is very fast but its range of validity is limited to small imperfection amplitudes and almost linear pre-buckling path. A second strategy was recently proposed by [28] and, by overcoming many drawbacks of the first formulation, it is recommended when the effects of the imperfection are more important. In this work, the geometrical imperfection $\tilde{u}$ is assumed to be a linear combination of modal displacement shapes ${\dot{u}}_{i}$ with combination factors $\tilde{\psi_{i}}$,

(29) $$\tilde{u}=\sum_{i=1}^{m}\tilde{\psi_{i}}{\dot{u}}_{i},\;||\tilde{u}\left|\right|\leq {\tilde{u}}_{max},$$

scaled in order to have an assigned maximum amplitude ${\tilde{u}}_{max}$ chosen, for example, from experimental measurements.

**The Global Convergent Method of Moving Asymptotes**. The optimal design problem is solved using a gradient-based optimizer, i.e., the Global Convergent Method of Moving Asymptotes (GCMMA) [33,38,39,40]. This algorithm devised for the optimization of objective functions requires a relatively high computational cost to evaluate the gradient and is characterized by many optimization variables. It is based on convex subsequent approximations of the objective function.

Constructing the gradient of the objective function with respect to the design parameters is not a simple task and is thus computed numerically. The *i*-th component of the gradient is evaluated according to a forward finite difference scheme as

(30) $$\nabla P_{i}\left(\alpha \right)\approx \frac{P\left(\alpha +de_{i}\right)-P\left(\alpha \right)}{d},$$

where *d* is a conveniently small finite difference and $e_{i}$ is a basis vector whose *i*th component is 1 while the others are zero.

Although the task of evaluating the objective function is extremely time-consuming when using the full discrete model, the efficiency of Koiter’s method allows its computational cost to be reduced to within an acceptable level. Moreover, since GCMMA generally converges with relatively few iterations, the number of gradient evaluations is small and the overall computational cost of the optimization is sustainable.

## 7. Numerical Results

In this section, the CNTs distribution is optimized in order to improve the nonlinear response under buckling. The three types of optimizations discussed in Section 3 are here summarized:

- OPT1: through-the-thickness optimization, i.e., variable through-the-thickness distribution of aligned CNTs across the shell mid-surface with assigned mid-surface value;
- OPT2: optimization across the mid-surface, i.e., the volume fraction of randomly oriented CNTs is kept constant through the thickness but can vary within the shell mid-surface with a constraint on the overall CNTs volume;
- OPT3: optimization of the orientation, i.e., the CNTs volume is assigned, the orientation of the CNTs can vary across the shell mid-surface but is constant through the thickness.

Three applications are considered. The first samples are an Euler beam and a simply supported plate in compression exhibiting a stable post-buckling behavior. In this case, the optimizations are expected to provide a higher buckling load and a less compliant post-buckling response. The last test regards a curved panel, whose post-buckling behavior can be either stable or unstable depending on the material distribution. This is a more significant test to show the formidable potential of a variable CNTs distribution in the design of thin nanocomposite structures.

We consider shells made of an isotropic polymeric matrix with Young modulus E=2.47 GPa and Poisson’s ratio ν=0.36. CNTs are considered as an isotropic elliptical inclusion with aspect ratio equal to 732, Young modulus E=970 GPa and Poisson’s ratio ν=0.1.

Two material descriptions over the mid-surface are considered: polynomials of order 4 and 9. Due to the problem symmetry, only the control points of a quarter of the structure are considered as independent variables, leading to 3×3 and 5×5 design control points, respectively, for the two descriptions. The number of optimization variables is 9 and 25 for the two cases, respectively.

For the OPT2 problem, the volume fraction bounds are set to $\phi_{C}^{min}=0.1\%$ and $\phi_{C}^{max}=15\%$ while the CNTs volume is given by $V_{C}=\phi_{C}^{*}V$.

It is worth noting that the effective CNTs volume fraction decreases by increasing $\phi_{C}$ according to [14] as shown in Table 1 due to CNTs agglomeration phenomena which are detrimental for the load transfer.

### 7.1. Nanocomposite Beam Under Compression

**Figure 2:**
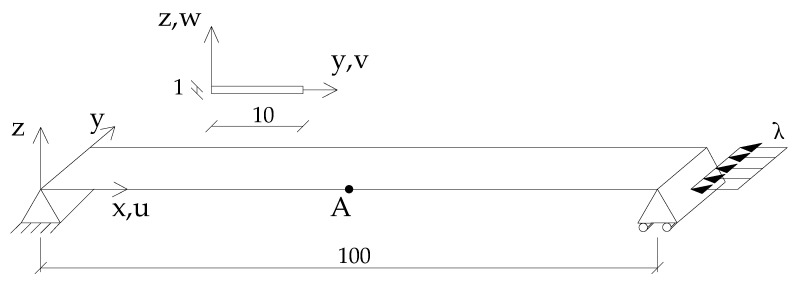
Euler beam: geometry (lengths in mm), loads and boundary conditions.

This test consists of a simply supported Euler beam under compression on two opposite ends as shown in Figure 2.

This simple test is considered to show the correctness of the proposed numerical approach. A single linearized buckling mode is used to construct the ROM of the Koiter method and the geometrical imperfection. The imperfection shape is scaled such that the maximum deviation ${\tilde{u}}_{max}$ is 0.1 of the shell thickness *h*. The deformation limit is set to v=2 mm with *v* the mean value of the flexural displacement.

**Figure 3:**
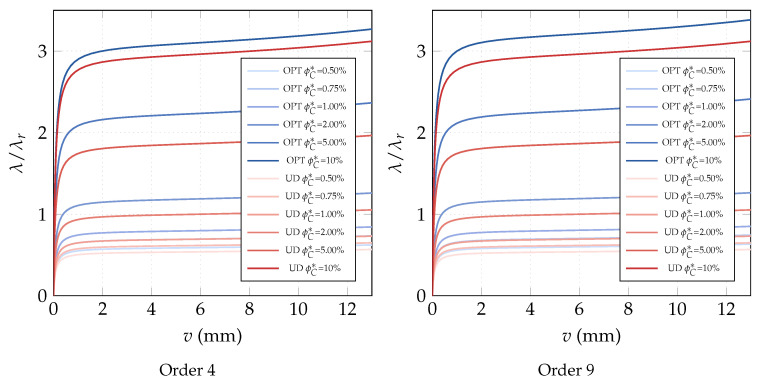
Euler nanocomposite beam: equilibrium paths for the optimal volume fraction distribution $\phi_{C}\left(\zeta_{1},\zeta_{2}\right)$ with assigned average value $\phi_{C}^{*}$ described by Bernstein polynomials of order 4 and 9 ($\lambda_{r}=0.0005313$ KN/mm).

**Figure 4:**
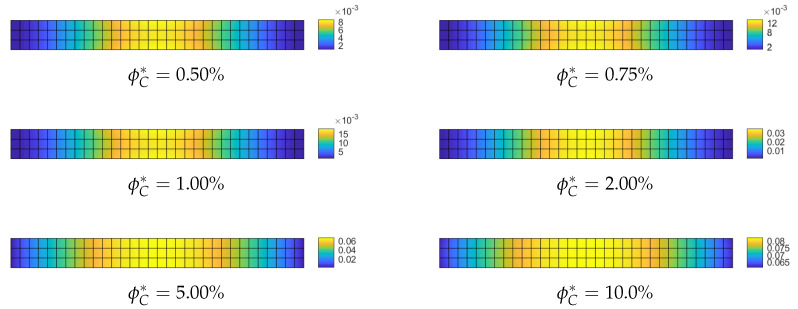
Euler nanocomposite beam: optimized volume fraction $\phi_{C}\left(\zeta_{1},\zeta_{2}\right)$ for assigned average value $\phi_{C}^{*}$ described by Bernstein polynomials of order 4.

**Figure 5:**
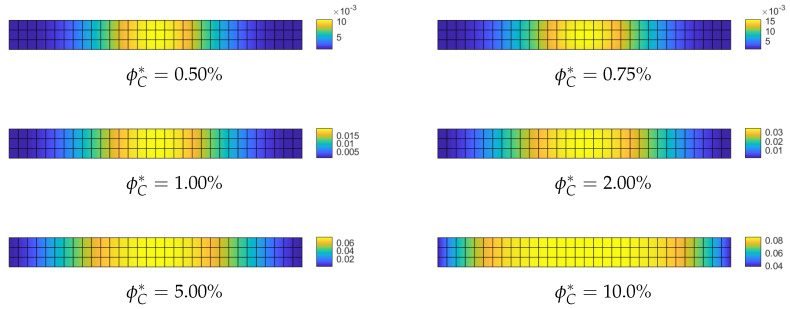
Euler nanocomposite beam: optimized volume fraction $\phi_{C}\left(\zeta_{1},\zeta_{2}\right)$ for assigned average value $\phi_{C}^{*}$ described by Bernstein polynomials of order 9.

**Optimization of the CNT volume fraction for random orientations**. The optimization problem OPT2 only is considered because OPT1 and OPT3 do not yield any improvement for this specific problem. The results of OPT2 are compared with the performance of a uniform distribution of $\phi_{C}$ (hereafter referred to as UD). Two polynomial orders are used to describe the distribution of $\phi_{C}$ across the surface: order 4 and order 9. First, we compare the linearized buckling loads reported in Table 2 for order 4. We note an increase of the first load for all average volume fractions $\phi_{C}^{*}$ and in particular for the case $\phi_{C}^{*}=5\%$ where the improvement reaches 20%. Similar considerations hold for the description with order 9 as shown in Table 3. A similar improvement is highlighted by a full nonlinear analysis in Figure 3. Finally, the optimal distribution of CNTs volume fraction is depicted in Figure 4 and Figure 5 for the two orders used to describe $\phi_{C}$ over the surface. As expected, it is possible to observe that, by comparison with the initial uniform distribution, the CNTs volume fraction is greater at the midspan of the beam and lower at the end sections in order to maximize the flexural stiffness. Moreover, it is worth noting that the effectiveness ratio of the CNTs decreases by increasing $\phi_{C}$ as shown in Table 1. This is the reason why the optimal distribution tends to be more uniform and equal to the maximum admissible fraction near the midspan as shown in Figure 4 and Figure 5 for high values of $\phi_{C}^{*}$.

### 7.2. Nanocomposite Plate under Compression

**Figure 6:**
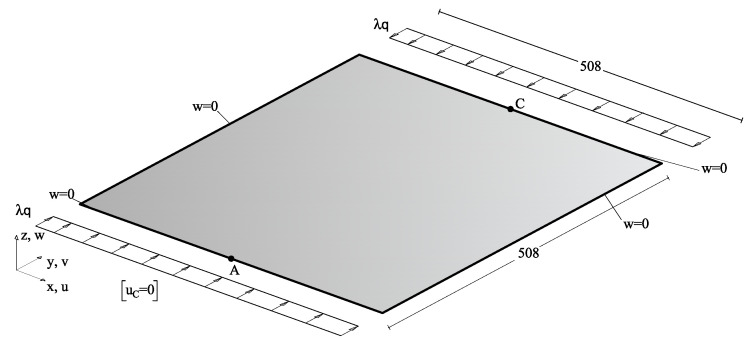
Square nanocomposite plate: geometry (lengths in mm), loads and boundary conditions.

This test deals with a simply supported square plate under compression on two opposite sides as shown in Figure 6.

The thickness was set to 5 mm while the span is 508 mm, and the uniform compression load was q=0.01 KN/mm.

A single linearized buckling mode is used to construct the ROM of the Koiter method and the geometrical imperfection. The imperfection shape is scaled such that the maximum deviation ${\tilde{u}}_{max}$ is 0.1h, with *h* being the shell thickness. The deformation limit is set to v=2 mm with *v* being the mean value of the out-of-plane displacement. Due to the symmetry of the test, OPT1 does not provide any improvement compared to a uniform volume fraction distribution and is omitted. Instead, the focus is on OPT2 and OPT3, respectively.

**Figure 7:**
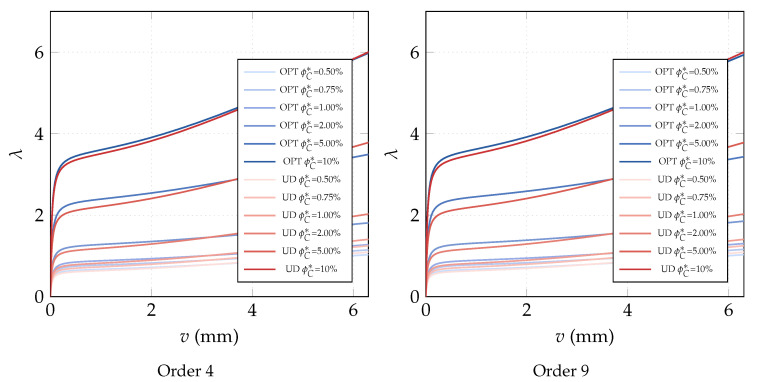
Square nanocomposite plate: equilibrium paths for the optimal volume fraction distribution $\phi_{C}\left(\zeta_{1},\zeta_{2}\right)$ with assigned average value $\phi_{C}^{*}$ described by Bernstein polynomials of order 4 and 9.

**Figure 8:**
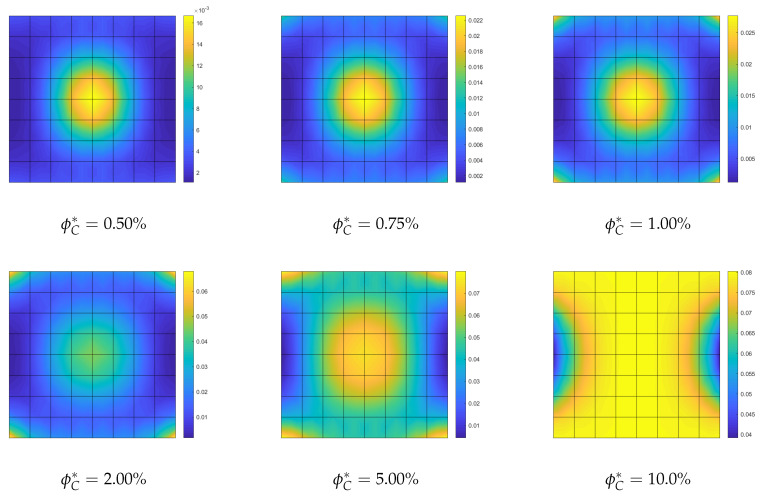
Square nanocomposite plate: optimized volume fraction distribution $\phi_{C}\left(\zeta_{1},\zeta_{2}\right)$ with assigned average value $\phi_{C}^{*}$ described by Bernstein polynomials of order 9.

**Optimization of the CNTs volume fraction for random orientations**. The optimization problem OPT2 is discussed here, making use of a comparison with the solution obtained for the uniform distribution of $\phi_{C}$ (UD). The linearized buckling loads are reported in Table 4 for the polynomial description of $\phi_{C}\left(\zeta_{1},\zeta_{2}\right)$ of order 4. We note a general but very slight increase of the first load for all average volume fractions $\phi_{C}^{*}$. Similar considerations hold for the parametrization of order 9 where the optimized solution is slightly better. The same considerations hold if we consider the full nonlinear paths shown in Figure 7. Finally, the optimal distribution of CNTs volume fraction is depicted in Figure 8 for 9th-order polynomials employed to describe $\phi_{C}$ across the surface. For the simply supported square plate, the use of a nonuniform distribution of randomly CNTs within the mid-surface does not yield significant improvements.

**Figure 9:**
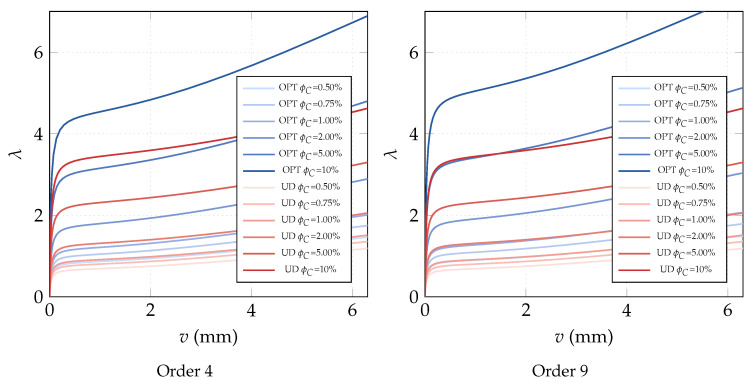
Square nanocomposite plate: equilibrium paths for the optimal CNTs orientation $\theta (\zeta_{1},\zeta_{2})$ described by Bernstein polynomials of order 4 and 9 for assigned volume fraction $\phi_{C}$.

**Figure 10:**
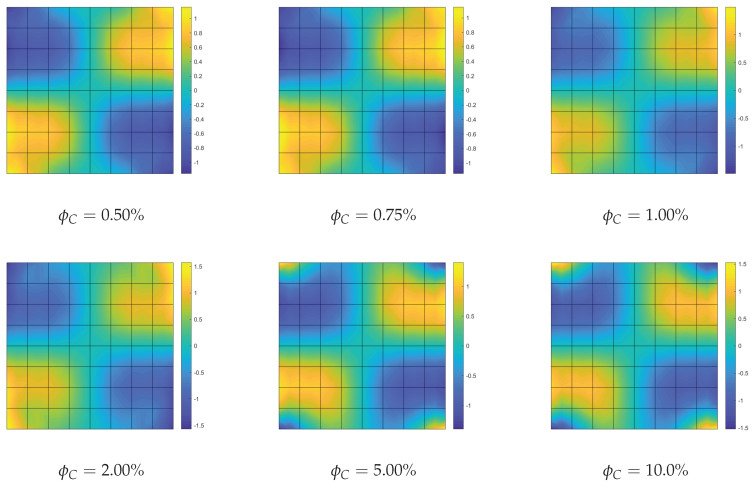
Square nanocomposite plate: optimal orientation $\theta (\zeta_{1},\zeta_{2})$ described by Bernstein polynomials of order 9 for assigned volume fraction $\phi_{C}$.

**Optimization of the CNTs orientation within the mid-surface**. The optimization problem OPT3 is investigated next. It consists in optimizing the orientation of the CNTs in the plate plane, while keeping the volume fraction constant. The results of OPT3 are compared with those corresponding to all CNTs uniformly aligned with the load direction (UD). The linearized buckling loads are reported in Table 5 for the polynomial parametrization of the orientation of order 4. It is possible to observe that the first buckling load is increased notably by the optimization and the improvement gets better with the volume fractions. Figure 9 with the full nonlinear path shows that a higher post-buckling stiffness can be obtained for the optimized solutions compared to the uniform orientation and an even better behavior is obtained with the parametrization of order 9. The results are completed with Figure 10 depicting the optimal orientation distribution of order 9.

### 7.3. Cylindrical Nanocomposite Panel under Compression

**Figure 11:**
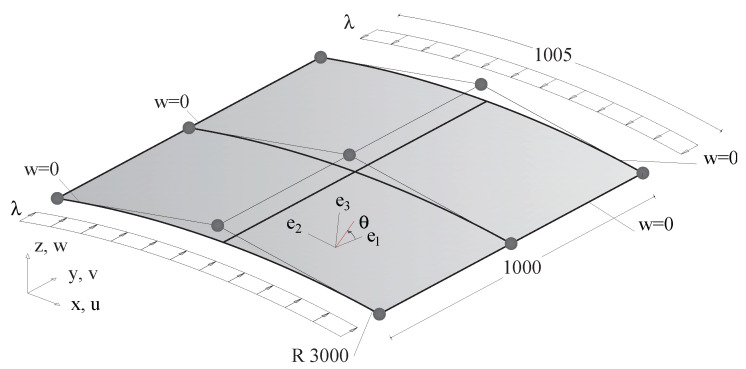
Cylindrical nanocomposite: geometry (lengths in mm), NURBS control grid, loading and boundary conditions.

The last test in Figure 11 features a cylindrical panel under compression. The structure presents a simple geometry but complex post-buckling behavior, involving multi-modal interactions with possible unstable path and significant imperfection sensitivity. This kind of panel was already subject to optimization for composite laminates by means of a stochastic optimizer [26,27]. Additionally, it was taken as a benchmark example to test the accuracy of the isogeometric solid-shell model [36].

The optimizations seek the material distributions that maximize the collapse load considering as deformation limit the axial displacement of the loaded section $v_{ls}=0.2$ mm. The maximum amplitude of the geometrical imperfection is ${\tilde{u}}_{x}=0.5\;h$. The first eight linearized buckling modes are used to construct the ROM of the Koiter method and the geometrical imperfection shape.

**Figure 12:**
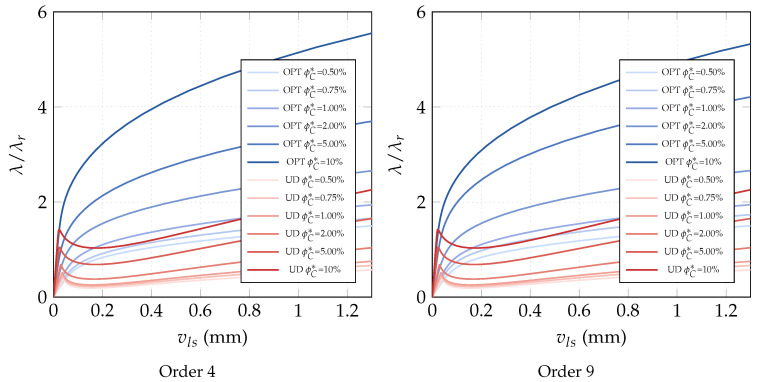
Cylindrical nanocomposite panel: equilibrium paths for the optimal through-the-thickness CNTs distribution with function $a(\zeta_{1},\zeta_{2})$ in Equation (20) described by Bernstein polynomials of order 4 and 9 for assigned volume fraction $\phi_{C}^{*}$ ($\lambda_{r}=0.006466$ KN/mm).

**Figure 13:**
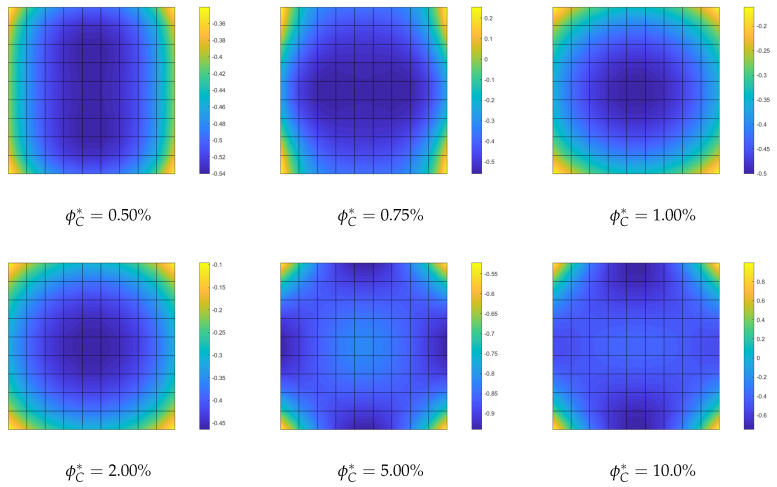
Cylindrical panel: optimized variability function $a[\zeta_{1},\zeta_{2}]$ in Equation (20) described by Bernstein polynomials of order 4 for assigned $\phi_{C}^{*}$.

**Through-the-thickness optimization of aligned CNTs volume fraction**. We start with the through-the-thickness optimization problem referred to as OPT1. The CNTs are aligned along the load direction. The results are compared with those obtained for a uniform through-the-thickness distribution (UD). First, Table 6 shows that the first linearized buckling load turns out to be almost unaffected by the optimization process. On the contrary, the full nonlinear analysis reported in Figure 12 shows how the optimization globally turns the behavior from unstable into stable using the same CNTs volume. The snap-through behavior is completely suppressed at the cost of a very slight stiffness reduction in the pre-critical range. Similar results in terms of equilibrium paths are obtained with a polynomial description of the function $a(\zeta_{1},\zeta_{2})$ of order 9. The shape of $a(\zeta_{1},\zeta_{2})$ is very similar for the two orders and is reported in Figure 13 for order 4.

**Figure 14:**
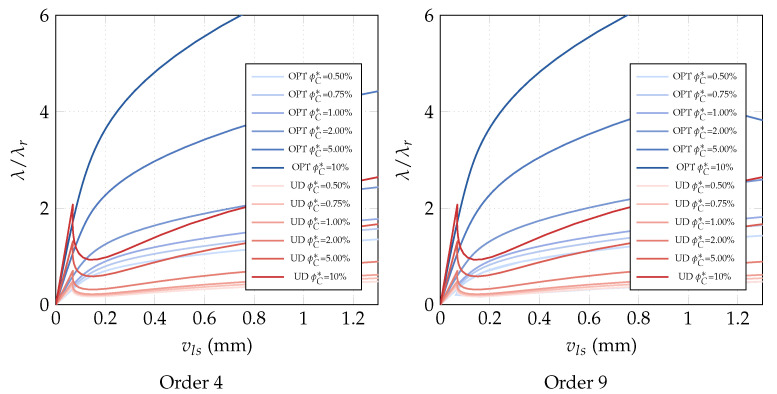
Cylindrical nanocomposite panel: equilibrium paths for the optimal volume fraction distribution $\phi_{C}\left(\zeta_{1},\zeta_{2}\right)$ with assigned average value $\phi_{C}^{*}$ described by Bernstein polynomials of order 4 and 9 ($\lambda_{r}=0.006466$ KN/mm).

**Figure 15:**
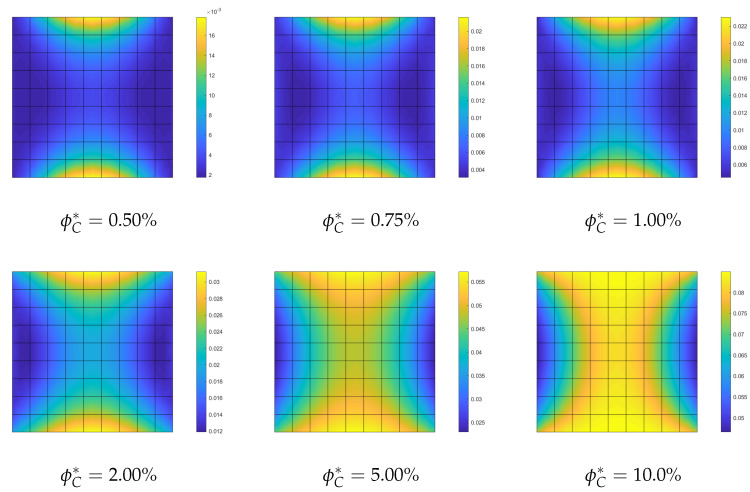
Cylindrical nanocomposite panel: optimized volume fraction distribution $\phi_{C}\left(\zeta_{1},\zeta_{2}\right)$ with assigned average value $\phi_{C}^{*}$ described by Bernstein polynomials of order 9.

**Optimization of CNTs volume fraction for random orientations**. The optimization problem OPT2 is considered next. The results are compared with the performance of a uniform distribution of randomly oriented CNTs (UD). First, we compare the linearized buckling loads reported in Table 7 for order 4. We note a general increase of the first load for all average volume fractions $\phi_{C}^{*}$ and, in particular, for the intermediate $\phi_{C}^{*}$ whose improvement is about 10%. Similar considerations hold for the parametrization of order 9 as shown in Table 8 where the optimized solution is also slightly improved. However, the great benefit of the variable volume fraction distribution is highlighted by a full nonlinear analysis in Figure 14. The unstable behavior of the uniform distribution is made stable by the optimal CNTs distribution using the same overall amount of CNTs. The slight stiffness reduction in the pre-critical range is compensated by the complete elimination of snap-through, at least in the range of interest. Similar results in terms of equilibrium paths are obtained with a polynomial description of $\phi_{C}\left(\zeta_{1},\zeta_{2}\right)$ of order 9. Indeed, the analysis leads to a very similar optimal distribution for the two orders, reported in Figure 15 for order 9.

**Figure 16:**
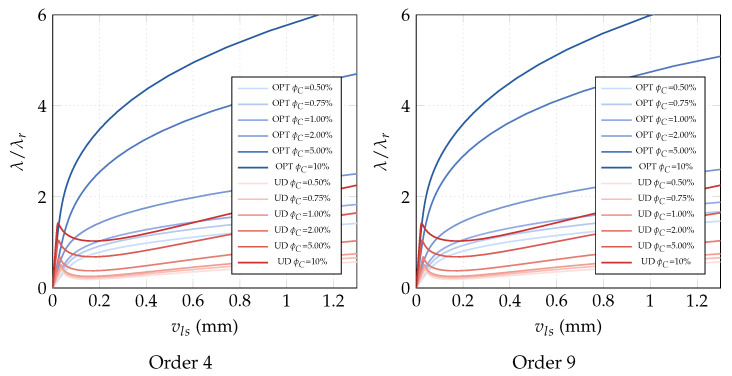
Cylindrical nanocomposite panel: equilibrium paths for the optimal CNTs orientation $\theta (\zeta_{1},\zeta_{2})$ described by Bernstein polynomials of order 4 and 9 for assigned volume fractions $\phi_{C}$ ($\lambda_{r}=0.006466$ KN/mm).

**Figure 17:**
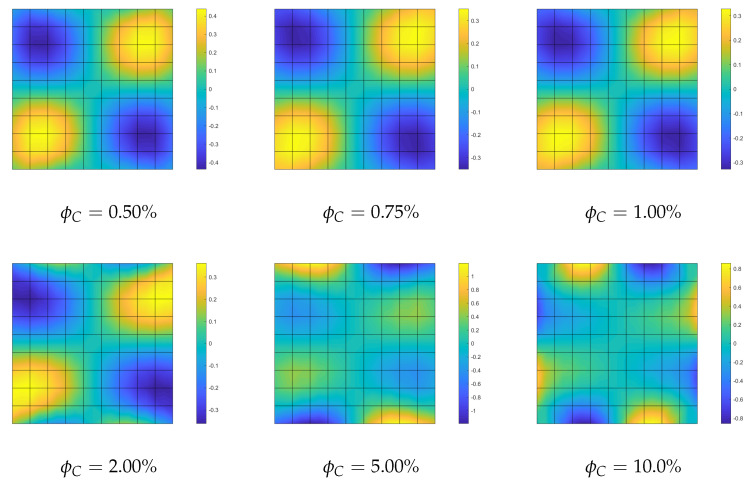
Cylindrical nanocomposite panel: optimized orientation $\theta (\zeta_{1},\zeta_{2})$ described by Bernstein polynomials of order 9 for assigned volume fraction $\phi_{C}$.

**Optimization of CNTs orientation**. The optimization problem OPT3 is finally discussed here. It consists of optimizing the CNTs orientation within the shell mid-surface, while keeping constant the volume fraction at each point of the structure. The results of OPT3 are compared with those obtained for uniformly aligned CNTs collinear with the load direction (UD). The linearized buckling loads are reported in Table 9 for the parametrized orientation distribution of order 4. It is possible to observe that the first buckling load becomes notably larger for high volume fractions, while it remains almost the same for low CNT contents. However, looking at Figure 16, the radical change of mechanical behavior is appreciable also for low volume fractions. The optimal CNTs distribution leads to the suppression of the snap-through instability, at the cost of initial stiffness reduction for low $\phi_{C}$, reduction that becomes negligible for higher volume fractions. Similar results are obtained with the parametrization of order 9. The results are completed with Figure 17 depicting the optimal orientation distribution of order 9.

## 8. Conclusions

This work tackled the new challenging problem of finding optimal distributions of the nanostructural reinforcement phase (here, carbon nanotubes) within polymeric composite panels subject to buckling and snap-through.

- A numerical strategy for the optimization of the buckling and postbuckling response of nanocomposite shells with variable CNTs distribution was proposed and investigated. The method is based on an integrated isogeometric framework that employs NURBS functions to describe the geometry and displacements while the optimization variables deal with the CNT distributions within the polymer hosting matrix.
- Various CNTs distributions were investigated either through the thickness or within the mid-surface for both aligned CNTs, aligned but varying within the surface or randomly oriented. The obtained through-the thickness distributions can be practically realized in multilayer nanocomposite structures since a continuous law can be reasonably approximated by piece-wise functions when the multilayers are considered sufficiently thin.
- The outcomes of extensive numerical tests have proved that the limit load can be largely improved for optimal CNTs distributions in the sense of strategically deploying the nanofibers where the maximum elastic stiffness fighting against the negative stiffness can be attained.
- Most importantly, it has been shown that shallow shells, which are dangerously prone to snap-through, can become globally stable if the CNTs are optimally distributed. This is a remarkable result on the global stability of nanocomposite shallow shells which, when properly designed, do not show any snap-through and thus can be safely employed in engineering applications. Mention must be made of the fact that these nanocomposite panels also show the additional advantage of exhibiting enhanced damping capability thanks to the CNT/polymer interfacial dissipation which makes these structures generally more stable against dynamic loads.
- This work has shown the potential of optimizing nonlinear structural behaviors using the unprecedented flexibility afforded by the CNTs nanoreinforcement which not only acts to shift the elastic loss of stability towards higher stresses, but can also either suppress snap-through or make the response less compliant in the postbuckling range. The next step of the research will be the optimal design of high-performance and lightweight vehicles, aerostructures and devices.

## Figures and Tables

**Table 1:** Effective volume fraction (in %).

nominal	0.50	0.75	1.00	2.00	5.00	10.0	15.0
effective	0.48	0.72	0.96	1.89	4.45	7.570	8.50

**Table 2:** Euler beam: linearized buckling loads for the optimal volume fraction distribution $\phi_{C}\left(\zeta_{1},\zeta_{2}\right)$ with assigned average value $\phi_{C}^{*}$ described by Bernstein polynomials of order 4 normalized with respect to $\lambda_{r}=0.0005313$ KN/mm.

Mode	0.50%		0.75%		1.00%		2.00%		5.00%		10.0%	
	OPT	UD	OPT	UD	OPT	UD	OPT	UD	OPT	UD	OPT	UD
1	0.5899	0.5386	0.6951	0.6163	0.7977	0.6936	1.1867	1.0000	2.2341	1.8662	3.1037	2.9634
2	2.1437	2.1621	2.4200	2.4737	2.6829	2.7843	3.7129	4.0141	7.3660	7.4911	12.027	11.894
3	4.8159	4.8906	5.4150	5.5955	5.9816	6.2982	8.1653	9.0798	15.715	16.944	26.985	26.904
4	8.6074	8.7516	9.6712	10.013	10.675	11.270	14.512	16.248	27.450	30.322	48.143	48.142

**Table 3:** Euler beam: linearized buckling loads for the optimal volume fraction distribution $\phi_{C}\left(\zeta_{1},\zeta_{2}\right)$ with assigned average value $\phi_{C}^{*}$ described by Bernstein polynomials of order 9 normalized with respect to $\lambda_{r}=0.0005313$ KN/mm.

Mode	0.50%		0.75%		1.00%		2.00%		5.00%		10.0%	
	OPT	UD	OPT	UD	OPT	UD	OPT	UD	OPT	UD	OPT	UD
1	0.5988	0.5386	0.7020	0.6163	0.8026	0.6936	1.1897	1.0000	2.2694	1.8662	3.2106	2.9634
2	2.0477	2.1621	2.3072	2.4737	2.5889	2.7843	3.6998	4.0141	6.8262	7.4911	12.350	11.894
3	4.7410	4.8906	5.2998	5.5955	5.8482	6.2982	8.0605	9.0798	14.120	16.944	27.260	26.904
4	8.4905	8.7516	9.4941	10.013	10.486	11.270	14.363	16.248	24.777	30.322	48.199	48.142

**Table 4:** Square plate: linearized buckling loads for the optimal volume fraction distribution $\phi_{C}\left(\zeta_{1},\zeta_{2}\right)$ with assigned average value $\phi_{C}^{*}$ described by Bernstein polynomials of order 4.

Mode	0.50%		0.75%		1.00%		2.00%		5.00%		10.0%	
	OPT	UD	OPT	UD	OPT	UD	OPT	UD	OPT	UD	OPT	UD
1	0.6736	0.6358	0.7835	0.7276	0.8892	0.8191	1.2947	1.1810	2.4128	2.2030	3.5968	3.4943

**Table 5:** Square nanocomposite plate: linearized buckling loads for the optimal CNTs orientation $\theta (\zeta_{1},\zeta_{2})$ described by Bernstein polynomials of order 4 for assigned volume fraction $\phi_{C}$.

Mode	0.50%		0.75%		1.00%		2.00%		5.00%		10.0%	
	OPT	UD	OPT	UD	OPT	UD	OPT	UD	OPT	UD	OPT	UD
1	0.8611	0.6907	1.0369	0.8038	1.2020	0.9131	1.7696	1.3199	3.1007	2.3369	4.5583	3.4909

**Table 6:** Cylindrical nanocomposite panel: linearized buckling loads for the optimal through-the-thickness CNTs distribution with function $a(\zeta_{1},\zeta_{2})$ in Equation (20) described by Bernstein polynomials of order 4 and assigned volume fraction $\phi_{C}^{*}$ normalized with respect to $\lambda_{r}=0.006466$ KN/mm.

Mode	0.50%		0.75%		1.00%		2.00%		5.00%		10.0%	
	OPT	UD	OPT	UD	OPT	UD	OPT	UD	OPT	UD	OPT	UD
1	0.6325	0.6065	0.7230	0.6878	0.7920	0.7605	1.0336	1.0000	1.4586	1.4978	1.9950	1.9873
2	0.7947	0.7861	0.9166	0.9108	1.0286	1.0283	1.4351	1.4507	2.1035	2.4574	3.3456	3.5521
3	1.1102	1.0811	1.2726	1.2461	1.4225	1.4029	1.9248	1.7974	2.8299	2.6611	3.7066	3.5924
4	1.3092	1.2268	1.4444	1.3339	1.5427	1.4354	1.9806	1.9787	2.9550	3.3660	4.6859	4.8864
5	1.3720	1.3669	1.6309	1.6260	1.8700	1.8737	2.7485	2.7820	4.2409	5.0083	6.9759	7.4824
6	1.5385	1.5380	1.8357	1.8323	2.1058	2.1160	3.1257	3.1646	4.8939	5.5607	7.6127	7.7939
7	2.2954	2.2719	2.5844	2.5079	2.8048	2.7305	3.6036	3.5502	5.1469	5.7560	8.2031	8.6383
8	2.3122	2.3043	2.6621	2.6583	2.9917	3.0021	4.2227	4.2762	6.3765	7.3119	10.0125	9.1880

**Table 7:** Cylindrical nanocomposite panel: linearized buckling loads for the optimal volume fraction distribution $\phi_{C}\left(\zeta_{1},\zeta_{2}\right)$ with assigned average value $\phi_{C}^{*}$ described by Bernstein polynomials of order 4 normalized with respect to $\lambda_{r}=0.006466$ KN/mm.

Mode	0.50%		0.75%		1.00%		2.00%		5.00%		10.0%	
	OPT	UD	OPT	UD	OPT	UD	OPT	UD	OPT	UD	OPT	UD
1	0.6075	0.5552	0.6942	0.6353	0.7832	0.7151	1.1173	1.0310	1.9557	1.9237	3.1329	3.0530
2	0.7684	0.7010	0.8776	0.8022	0.9892	0.9030	1.4251	1.3019	2.4889	2.4290	3.9793	3.8548
3	1.0120	1.0087	1.1678	1.1543	1.3151	1.2994	1.8771	1.8736	3.3529	3.4952	5.3862	5.5450
4	1.1833	1.1107	1.3590	1.2708	1.5277	1.4305	2.2466	2.0625	3.9364	3.8483	6.2432	6.1075
5	1.2680	1.2401	1.4611	1.4190	1.6476	1.5974	2.3504	2.3032	4.1880	4.2968	6.7559	6.8168
6	1.3703	1.4200	1.5833	1.6249	1.7772	1.8292	2.5711	2.6374	4.6157	4.9201	7.3073	7.8052
7	1.9120	1.7370	2.1885	1.9875	2.4664	2.2372	3.5623	3.2256	6.2360	6.0183	9.9263	9.5510
8	2.0283	1.9699	2.3364	2.2541	2.6371	2.5374	3.7589	3.6584	6.6961	6.8254	10.807	10.83039

**Table 8:** Cylindrical nanocomposite panel: linearized buckling loads for the optimal volume fraction distribution $\phi_{C}\left(\zeta_{1},\zeta_{2}\right)$ with assigned average value $\phi_{C}^{*}$ described by Bernstein polynomials of order 9 normalized with respect to $\lambda_{r}=0.006466$ KN/mm.

Mode	0.50%		0.75%		1.00%		2.00%		5.00%		10.0%	
	OPT	UD	OPT	UD	OPT	UD	OPT	UD	OPT	UD	OPT	UD
1	0.6237	0.5552	0.7167	0.6353	0.8029	0.7151	1.1353	1.0310	2.0376	1.9237	3.1647	3.0530
2	0.7799	0.7010	0.8936	0.8022	1.0059	0.9030	1.4267	1.3019	2.5885	2.4290	4.0217	3.8548
3	1.0178	1.0087	1.1699	1.1543	1.3147	1.2994	1.8990	1.8736	3.4311	3.4952	5.4352	5.5450
4	1.1623	1.1107	1.3317	1.2708	1.5204	1.4305	2.1848	2.0625	4.0413	3.8483	6.3539	6.1075
5	1.2778	1.2401	1.4679	1.4190	1.6554	1.5974	2.3892	2.3032	4.3172	4.2968	6.8085	6.8168
6	1.3526	1.4200	1.5470	1.6249	1.7422	1.8292	2.5344	2.6374	4.6328	4.9201	7.4096	7.8052
7	1.9405	1.7370	2.2259	1.9875	2.5083	2.2372	3.5554	3.2256	6.4522	6.0183	10.0560	9.5510
8	2.0844	1.9699	2.3961	2.2541	2.6806	2.5374	3.8509	3.6584	6.9260	6.8254	10.8735	10.8303

**Table 9:** Cylindrical nanocomposite panel: linearized buckling loads for the optimal CNTs orientation $\theta (\zeta_{1},\zeta_{2})$ described by Bernstein polynomials of order 4 for assigned volume fraction $\phi_{C}$ normalized with respect to $\lambda_{r}=0.006466$ KN/mm.

Mode	0.50%		0.75%		1.00%		2.00%		5.00%		10.0%	
	OPT	UD	OPT	UD	OPT	UD	OPT	UD	OPT	UD	OPT	UD
1	0.5956	0.6065	0.6710	0.6878	0.7368	0.7605	0.9519	1.0000	1.6582	1.4978	2.2547	1.9873
2	0.7753	0.7861	0.8947	0.9108	1.0060	1.0283	1.4116	1.4507	2.5809	2.4574	3.4228	3.5521
3	1.0966	1.0811	1.2574	1.2461	1.4100	1.4029	1.7751	1.7974	2.8041	2.6611	3.6373	3.5924
4	1.2540	1.2268	1.3588	1.3339	1.4587	1.4354	1.9597	1.9787	3.5398	3.3660	4.7805	4.8864
5	1.3938	1.3669	1.6453	1.6260	1.8821	1.8737	2.7433	2.7820	4.8703	5.0083	6.7610	7.4824
6	1.5363	1.5380	1.8221	1.8323	2.0941	2.1160	3.0839	3.1646	5.5986	5.5607	7.5653	7.7939
7	2.2102	2.2719	2.5331	2.5079	2.7802	2.7305	3.6117	3.5502	5.8414	5.7560	7.8166	8.6383
8	2.3373	2.3043	2.6906	2.6583	3.0311	3.0021	4.2693	4.2762	7.3583	7.3119	9.7666	9.1880
